# Mortality and Neurodevelopmental Outcome in an Italian Cohort of Very Low Birth Weight Infants

**DOI:** 10.1111/apa.70292

**Published:** 2025-08-27

**Authors:** Licia Lugli, Luca Bedetti, Marisa Pugliese, Isotta Guidotti, Gina Ancora, Giancarlo Gargano, Serafina Perrone, Agostina Solinas, Mario Motta, Luigi Tommaso Corvaglia, Alessandra Sansavini, Marcello Stella, Riccardo Cuoghi Costantini, Antonella DiCaprio, Elisa Della Casa Muttini, Maria Federica Roversi, Natascia Bertoncelli, Piero Catenazzi, Elisa Ballardini, Sara Grandi, Sabrina Moretti, Daniela Turoli, Arianna Aceti, Silvia Braibanti, Anna Insalaco, Camilla Migliozzi, Monica Fumagalli, Fabrizio Ferrari, Alberto Berardi

**Affiliations:** ^1^ Neonatal Intensive Care Unit University Hospital of Modena Modena Italy; ^2^ Psychology Unit University Hospital of Modena and Reggio Emilia Modena Italy; ^3^ Neonatal Intensive Care Unit Infermi Hospital of Rimini Rimini Italy; ^4^ Neonatal Intensive Care Unit Azienda Unità Sanitaria Locale‐IRCCS Reggio Emilia Italy; ^5^ Neonatal Intensive Care Unit University Hospital of Parma Parma Italy; ^6^ Neonatal Intensive Care Unit University Hospital of Ferrara Ferrara Italy; ^7^ Neonatal Intensive Care Unit Maggiore Hospital of Bologna Bologna Italy; ^8^ Department of Medical and Surgical Sciences University of Bologna Bologna Italy; ^9^ Neonatal Intensive Care Unit IRCCS AOUBO Bologna Italy; ^10^ Department of Psychology “Renzo Canestrari” University of Bologna Bologna Italy; ^11^ Neonatal Intensive Care Unit Bufalini Hospital of Cesena Cesena Italy; ^12^ Statistics Unit, Department of Medical and Surgical Sciences for Mother, Child and Adult University of Modena and Reggio Emilia Modena Italy; ^13^ Neonatal Intensive Care Unit Villa dei Fiori Hospital Napoli Italy; ^14^ Italian Neonatal Network University of Milan Milan Italy; ^15^ Department of Medical and Surgical Sciences of Mothers, Children and Adults, Pediatric Postgraduate School University of Modena and Reggio Emilia Modena Italy; ^16^ Neonatal Intensive Care Unit, Fondazione IRCCS Ca' Granda Ospedale Maggiore Policlinico University of Milan Milan Italy

**Keywords:** neonatal care, neurodevelopmental outcome, preterm, survival, very low birth weight

## Abstract

**Aim:**

Preterm infants face high risks of mortality and neurodevelopmental impairment. We aimed to evaluate the outcomes in an Italian cohort of very low birth weight infants.

**Methods:**

This multicenter prospective study included very low birth weight infants born in Italy between 2016 and 2020. Severe functional disability was defined as cognitive impairment, cerebral palsy, blindness or deafness.

**Results:**

A total of 1381 patients were enrolled (median gestational age: 29.1 weeks; males: 678) and 136 (9.8%) died. Multivariate analysis identified gestational age (OR 0.65), prenatal steroids (OR 0.36), magnesium sulphate (OR 0.48), advanced resuscitation (OR 4.04) and admission body temperature (OR 0.84) as predictors of mortality. A total of 802 (65.5%) completed the 24‐month neurodevelopmental follow‐up. Severe functional disability was observed in 71/802 (8.9%) infants, including neuropsychological impairment (59.1%), cerebral palsy (4.2%), deafness (1%) and blindness (0.2%). Multivariate analysis identified male gender (OR 1.84), advanced resuscitation (OR 1.92), periventricular leukomalacia (OR 9.94) and periventricular‐intraventricular haemorrhage (OR 1.74) as predictors of severe functional disability.

**Conclusion:**

Mortality and neurodevelopmental impairments in very low birth weight infants were associated with gestational age and neonatal complications. Early interventions, including prenatal steroids, magnesium sulphate and improved neonatal care, may enhance survival and outcomes.

**Abbreviations**
Bayley‐III: Bayley Scales of Infant and Toddler Development
BPD: bronchopulmonary dysplasia
CI: confidence intervals
FD: functional disability
GA: gestational age
GMDS‐R: Griffiths Mental Developmental Scales
NEC: necrotizing enterocolitis
NICU: neonatal intensive care unit
IQR: interquartile range
OR: odd ratio
PDA: patent ductus arteriosus
PIH: periventricular‐intraventricular haemorrhage
PVL: periventricular leukomalacia
REDCap: Research Electronic Data Capture
SD: standard deviation
VLBW: very low birth weight

**Summary**

- Very low birth weight infants face a high risk of death and neurodevelopmental impairment, highlighting the need for outcome data to guide clinical care.
- In this Italian multicentre prospective study, 9.8% of infants died, and 8.9% of the survivors had severe functional disabilities.
- The results support the role of prenatal interventions and improved neonatal care in enhancing survival and long‐term outcomes in this vulnerable group.

## 1 Introduction

The burden of neurodevelopmental disabilities, including cognitive, motor and sensory impairments, remains a major concern associated with preterm birth. Understanding the factors influencing both survival and developmental outcomes is crucial for optimising neonatal care strategies and providing informed prognostic counselling to families. Very low birth weight (VLBW) infants, defined as those weighing less than 1500 g at birth, represent a highly vulnerable population with increased risks of mortality and long‐term neurodevelopmental impairments [1, 2, 3, 4, 5, 6].

Italy, like many other high‐income countries, has witnessed improvements in perinatal and neonatal intensive care practices. However, variations in clinical management may contribute to differences in outcomes. Identifying key predictors of mortality and long‐term impairments in VLBW infants is crucial for optimising care strategies and ensuring equitable health interventions. In addition, data on mortality and disability can aid in prenatal counselling and ensure that families receive accurate information and support during neonatal intensive care unit (NICU) hospitalisation and follow‐up care [3, 6, 7, 8, 9].

A previous study, named *Neurological Outcome of Premature Infants 2* or Neuroprem 2, evaluated outcomes in very low birth weight infants in an Italian cohort [7]. Compared to Neuroprem 2, the present study includes a larger population, covers a longer study period, and aims to assess both mortality and neurodevelopmental outcomes. By analysing perinatal and neonatal factors, we seek to identify critical determinants that influence survival and developmental trajectories.

Our findings may contribute to the ongoing efforts in enhancing neonatal care and support strategies tailored to this high‐risk population.

This multicenter prospective study aimed to evaluate neonatal mortality, neurodevelopmental outcomes, and their predictors in a cohort of very low birth weight preterm infants born between 2016 and 2020.

## 2 Methods

This prospective, observational study was conducted by the Neuroprem Network, which comprised nine NICUs. Established in 2015, the Network developed the study protocol and defined the data collection methods. Data were collected using the Research Electronic Data Capture (REDCap), a secure, web‐based software platform developed and maintained by Vanderbilt University (Nashville, Tennessee, United States of America). Collected data included demographics, clinical information, and neurodevelopmental outcomes, which were recorded using a customised REDCap form.

Various perinatal data were analysed, including birth weight, gestational age (GA), delivery site and mode, gender, prenatal exposure to steroids and magnesium sulphate, chorioamnionitis, admission temperature, advanced neonatal resuscitation (delivery room intubation) and Apgar score. Moreover, neonatal information was evaluated, including sepsis, mechanical ventilation, patent ductus arteriosus (PDA) treatment, necrotising enterocolitis (NEC), bronchopulmonary dysplasia (BPD), cerebral lesions, retinopathy of prematurity and breastfeeding at discharge [7, 10].

Each centre entered the data, while a coordinating centre ensured completeness and accuracy through periodic communication and on‐site support when needed. Perinatal data and outcomes were regularly discussed during network meetings.

### 2.1 Study Population

VLBW infants born between January 1st, 2016 and December 31st, 2020, were enrolled and categorised into three groups based on gestational age: group one (≤ 28 weeks of gestation), group two (28–31 weeks of gestation) and group three (≥ 32 weeks of gestation). Neurodevelopmental outcome until corrected age of 24 months was assessed among survivors. Infants with genetic abnormalities or major malformations were excluded.

### 2.2 Neurodevelopmental Follow Up

Neurodevelopmental assessment was conducted by a multidisciplinary team, including a neonatologist, a developmental psychologist, a child physiotherapist and a paediatric neurologist. Depending on the local protocol, the Griffiths Mental Developmental Scales (GMDS‐R, 1996) or the Bayley Scales of Infant and Toddler Development III version (Bayley III, 2006) were used [7, 11, 12].

The GMDS‐R provides a global development quotient of infants' abilities and five subscale quotients (locomotor; eye and hand coordination; personal and social; hearing and language; cognitive performance). The Bayley III provides standardised composite scores for each of the assessed domains: cognitive, fine and gross motor, receptive and expressive language, adaptive domains [11, 12].

### 2.3 Definitions

Vermont Oxford Network definitions were adopted for neonatal data, like: [10]

- NEC: if diagnosed at surgery, at postmortem examination, or with clinical (at least one of the following clinical signs: bilious gastric aspirate or emesis, abdominal distension or discoloration, occult or gross blood in stool) and diagnostic imaging (at least one of the following findings: pneumatosis intestinalis, hepato‐biliary gas, pneumoperitoneum).
- Retinopathy of prematurity: if retinopathy of prematurity > 2 stage was present.
- BPD: was defined by the need for oxygen at 36 weeks of gestation.
- Sepsis: if a bacterial pathogen was recovered from a blood culture.
- Early‐onset sepsis (EOS): if a bacterial pathogen was recovered from a blood culture obtained on Days 1, 2 or 3 of life.
- Late‐onset sepsis (LOS): if a bacterial pathogen is recovered from a blood culture obtained after Day 3 of life.
- PDA was defined as the presence of left to right or bidirectional ductal shunt on doppler ultrasound and at least two of the following findings are present: hyperdynamic precordium, bounding pulses, wide pulse pressure, pulmonary vascular congestion, cardiomegaly or both.
- Periventricular leukomalacia (PVL): there must be multiple small periventricular cysts identified on a cranial ultrasound at any time.
- Periventricular‐intraventricular haemorrhage (PIH) indicates whether the infant has a > 2 grade periventricular‐intraventricular haemorrhage on or before Day 28 of life.
- Chorioamnionitis was defined by the presence of at least two of the following: elevated maternal C‐reactive protein, leukocytosis, fever > 38°C, malodorous amniotic fluid or maternal/foetal tachycardia. Neonatal sepsis was diagnosed based on a positive blood culture.
- Mechanical ventilation: conventional or high frequency ventilation through an endotracheal tube or tracheostomy at any time after leaving the delivery room/initial resuscitation area.

Other definitions:

- Severe functional disability (severe FD) was defined as at least one of the following: cerebral palsy, global developmental quotient or Bayley III cognitive score < 2 SD, bilateral blindness, bilateral deafness requiring hearing aids or implants or epilepsy.
- Cerebral palsy was defined as a permanent disorder of movement and posture causing activity limitations attributed to non‐progressive disturbances that occurred in the developing brain. The classification included spastic cerebral palsy (monoparesis, hemiparesis, triparesis, tetraparesis, diplegia) and extrapyramidal (dyskinetic) syndromes [13]. The rate and type of cerebral palsy were evaluated [13].
- Moderate functional disability (moderate FD) was defined as unilateral blindness or Bayley III cognitive composite score/GMDS‐R global development quotient ≤ 1 DS or moderate neuromotor abnormalities like abnormal tone pattern or clumsiness.

### 2.4 Statistical Analysis

Statistical analyses were performed using Stata Direct Statistical Software version 18 (StataCorp LP, United State of America). Continuous variables were expressed as means and standard deviation (SD) or medians and interquartile ranges (IQR), and categorical variables as frequencies and percentages. Differences between groups were assessed using the chi‐square test for categorical variables and the Mann–Whitney *U* test for continuous variables. Univariate and multivariate logistic regression analyses were performed to identify factors associated with death or severe FD. Multicollinearity was assessed using correlation coefficients and variance inflation factors. Odds ratios (OR) with 95% confidence intervals (CI) were calculated. Statistical significance was set at *p* < 0.05.

### 2.5 Ethics

The study was approved by the Emilia Romagna Ethics Committee (protocol 205/2015, n 4818). Written informed consent to participate in this study was provided by the participants' parents.

## 3 Results

**FIGURE 1:**
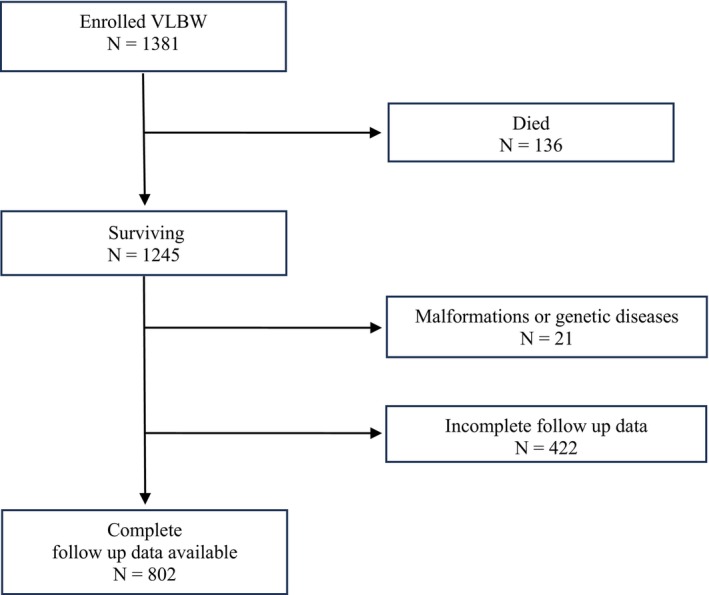
Flow chart of the study population.

We enrolled 1381 VLBW infants (median GA: 29.1 weeks, IQR 27–31; median birth weight: 1095 g, IQR 832–1350). Of these, 136 infants (9.8%) died before hospital discharge, within the term‐corrected age. Among the 1245 surviving patients, 21 (1.7%) were excluded because of major malformations or genetic anomalies. Of the remaining 1224 patients, 802 infants (65.5%) completed the 24‐month neurodevelopmental follow‐up (Figure 1).

Table 1 summarises survival data and neurodevelopmental outcomes for the overall patient population, as well as for the three groups stratified by GA.

**TABLE 1:** Survival, severe and moderate functional disability among three groups of different gestational ages.

	Group 1	Group 2	Group 3	All
	*N* = 639 (46.3%)	*N* = 479 (34.7%)	*N* = 263 (19%)	*N =* 1381
Infants who died	124 (19.4%)	12 (2.5%)	0 (0)	136 (9.8%)
Infants who survived	515 (80.6%)	467 (97.5%)	263 (100%)	1245 (90.2%)
Excluded infants^a^	18 (3.5%)	2 (0.4%)	1 (0.7%)	21 (1.7%)
Survivors who were followed up	260 (52.3%)	389 (83.6%)	153 (58.4%)	802 (65.5%)^a^
SFD	43 (16.5%)	21 (5.4%)	7 (4.6%)	71 (8.9%)
MFD	48 (18.5%)	30 (7.7%)	7 (4.6%)	85 (10.6%)
No disability	169 (65.0%)	338 (86.9%)	139 (90.8%)	646 (80.5%)

*Note:* Group 1: ≤ 28 weeks' gestation; Group 2: > 28–31 weeks' gestation; Group 3: ≥ 32 weeks' gestation.

Abbreviations: MFD, moderate functional disability; SFD, severe functional disability.

^a^ Excluded because of malformation or genetic disease.

### 3.1 Neonatal Mortality

**FIGURE 2:**
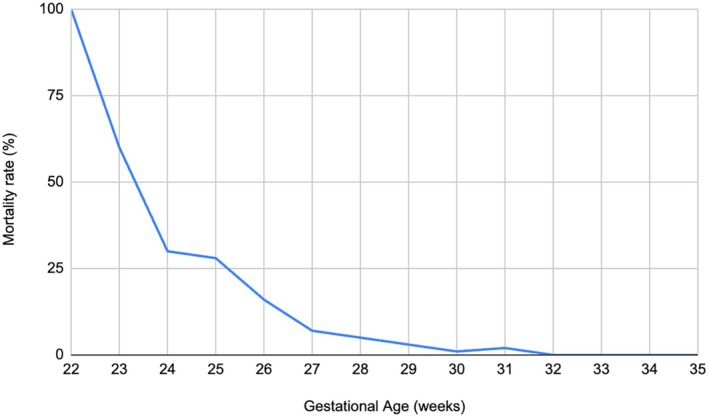
Mortality rates according to gestational age at birth.

Figure 2 and Figure S1 show the mortality rates in relation to GA. Surviving infants were more likely to have been exposed to prenatal steroids and magnesium sulphate. Infants who died had lower GA and lower body temperatures at admission; they were more often delivered after chorioamnionitis and required more advanced neonatal resuscitation. Patients who died had higher rates of sepsis, were more frequently mechanically ventilated, and showed a higher incidence of PIH (Table 2). Several variables were associated with death in the univariate logistic regression analysis (Table 3). Mechanical ventilation was not included among the predictors, as it can contribute to BPD and death, but may also reflect the severity of the underlying disease that itself leads to death. Absence of prenatal steroid or magnesium sulphate exposure, GA, advanced neonatal resuscitation and low body temperature at admission were independently associated with death, as shown by multivariate analysis.

**TABLE 2:** Neonatal characteristics among surviving and non‐surviving infants.

	Non survivors	Survivors	*p*
	*N* = 136	*N* = 1245	
Birth weight, median (IQR), g	670 (550–858)	1150 (895–1375)	**< 0.001**
Gestational age, median (IQR), weeks	25 (23.7–26.6)	29.4 (27.6–31.4)	**< 0.001**
Outborn, *n* (%)	9 (6.6)	46 (3.7)	0.093
Body temperature on admission, median (IQR), °C	35.8 (35–36.2)	36.1 (35.8–36.5)	**< 0.001**
Multiple gestation, *n* (%)	36 (26.5)	397 (31.9)	0.164
Advanced neonatal resuscitation, *n* (%)	101 (74.3)	278 (22.3)	**< 0.001**
Apgar 5th min, median (IQR)	7 (5–8)	9 (8–9)	**< 0.001**
Caesarean section, *n* (%)	61 (44.8)	264 (21.2)	**< 0.001**
Male gender, *n* (%)	77 (56.6)	601 (48.38)	0.089
Prenatal steroid exposure, *n* (%)	76 (55.9)	1121 (90.0)	**0.041**
Prenatal magnesium sulphate exposure, *n* (%)	22 (16.2)	441 (35.4)	**< 0.001**
Chorioamnionitis, *n* (%)	65 (47.8)	284 (22.8)	**< 0.001**
All sepsis, *n* (%)	39 (28.7)	121 (9.7)	**< 0.001**
EOS, *n* (%)	10 (7.4)	25 (2.0)	**< 0.001**
Mechanical ventilation, *n* (%)	131 (96.3)	432 (34.7)	**< 0.001**
PIH, *n* (%)	87 (63.9)	501 (40.2)	**< 0.001**
PDA, *n* (%)	82 (60.3)	441 (35.4)	**< 0.001**
PDA pharmacological treatment, *n* (%)	42 (30.8)	261 (20.9)	**0.006**
NEC, *n* (%)	16 (11.7)	39 (3.1)	**< 0.001**
NEC surgical treatment, *n* (%)	14 (10.3)	37 (3.0)	**< 0.001**

*Note:* The value in bold are statistically significant (*p* < 0.05).

Abbreviations: BPD, bronchopulmonary dysplasia; EOS, early‐onset sepsis; NEC, necrotizing enterocolitis; PDA, patent ductus arteriosus; PIH, periventricular–intraventricular haemorrhage; PVL, periventricular leukomalacia; ROP, retinopathy of prematurity.

**TABLE 3:** Uni‐ and multivariate analyses of factors associated with death.

	Univariate model			Multivariate model		
	OR	95% CI	*p*	OR	95% CI	*p*
Birth weight	0.99	0.99–1.00	**< 0.001**			
Gestational age	0.51	0.46–0.57	**< 0.001**	0.65	0.59–0.80	**< 0.001**
Outborn	1.86	0.89–3.90	**< 0.001**			
Body temperature admission	0.74	0.62–0.88	**< 0.001**	0.84	0.70–0.95	**0.008**
Multiple gestation	0.75	0.51–1.12	0.157			
Advanced neonatal resuscitation	16.43	10.08–26.79	**< 0.001**	4.04	1.13 4.41	**0.0210**
Apgar 5th min	0.90	0.79–1.03	0.091			
Caesarean delivery	2.96	2.06–4.27	**< 0.001**			
Male gender	1.36	0.95–1.95	0.089			
Prenatal steroid exposure	0.33	0.21–0.53	**< 0.001**	0.36	0.19–0.65	**0.0010**
Prenatal magnesium sulphate exposure	0.32	−0.20– 0.52	**< 0.001**	0.48	0.42–0.97	**0.0025**
Chorioamnionitis	3.21	2.22–4.64	**< 0.001**			
Sepsis	4.56	2.97–7.00	**< 0.001**			
EOS	4.17	2.21–7.88	**< 0.001**			
Periventricular–intraventricular haemorrhage	3.21	2.17–4.77	**< 0.001**			
PDA	3.25	2.21–4.76	**< 0.001**			
PDA pharmacological treatment	1.53	1.12–2.11	**0.008**			
NEC	2.50	1.54–4.08	**< 0.001**			
NEC surgical treatment	3.36	2.12–5.31	**< 0.001**			
Surfactant administration	10.85	6.06–19.42	**< 0.001**			

*Note:* The value in bold are statistically significant (*p* < 0.05).

Abbreviations: BPD, bronchopulmonary dysplasia; EOS, early‐onset sepsis; NEC, necrotizing enterocolitis; PDA, patent ductus arteriosus; PIH, periventricular–intraventricular haemorrhage; PVL, periventricular leukomalacia; ROP, retinopathy of prematurity.

### 3.2 Neurodevelopmental Outcome

Data on neurodevelopmental outcome were available for 802 of 1224 (65.5%) infants. The median GA was 29.2 weeks with an IQR of 27–31 weeks, and the median birth weight was 1138 g with an IQR of 890–1366 g. Infants who completed the 24‐month neurodevelopmental follow‐up were more likely to be inborn, delivered after a single pregnancy, and more exposed to prenatal magnesium sulphate and postnatal surfactant administration (*p* < 0.01). Infants who completed follow‐up and those who did not were comparable in terms of GA, birth weight and rate of cerebral lesions, such as PIH and PVL.

**FIGURE 3:**
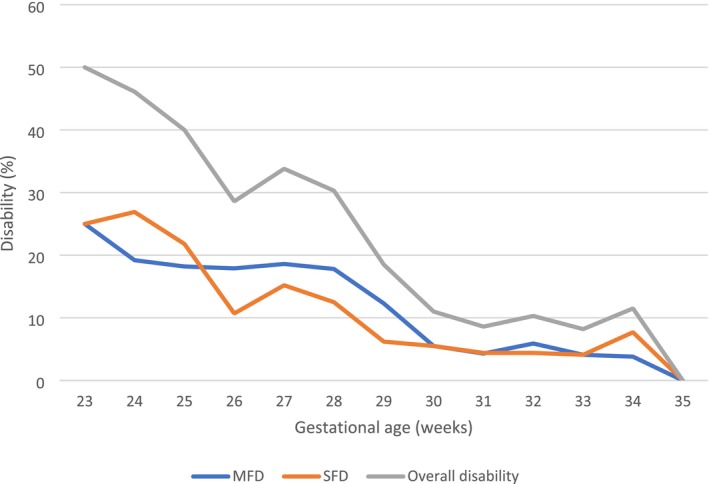
Moderate, severe and overall functional disability according to gestational age at birth. MFD, moderate functional disability; SFD, severe functional disability. Overall disability stands for the sum of moderate and severe disability.

**FIGURE 4:**
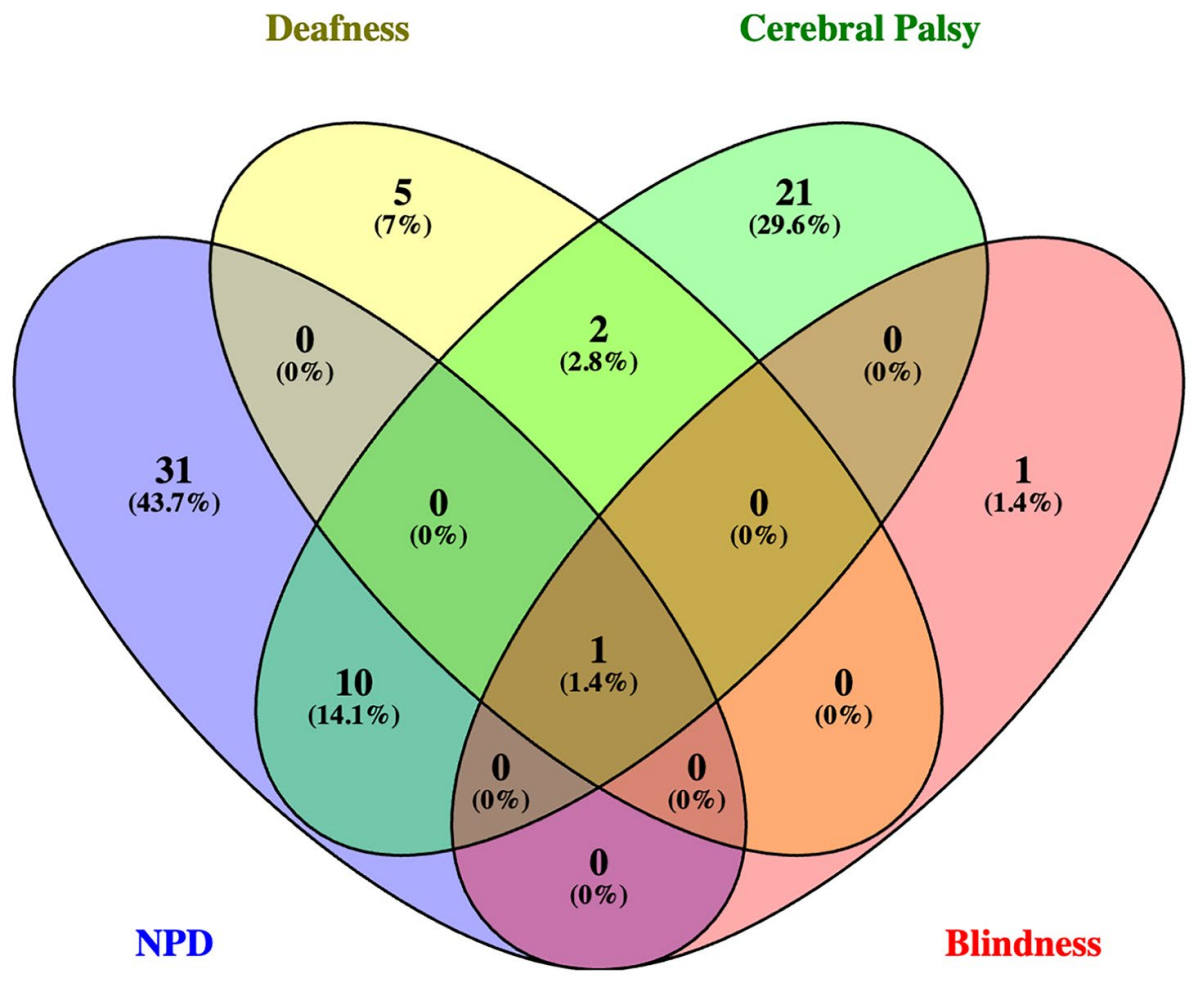
Severe functional disability Venn diagram. NPD, neuropsychological deficiencies (BSDI III cognitive composite score < 2 DS or a GMDS‐R GQ < 2 DS).

Table 4, Figure 3, and Figure S2 present data on severe, moderate and overall disability in relation to GA. Severe FD occurred in 71/802 infants (8.9%). Neuropsychological deficiency was found in 42/71 cases (59.1%): Bayley III cognitive composite score < 2 SD in 16 patients and GMDS‐R global development quotient < 2 SD in 26 patients. Cerebral palsy was diagnosed in 34/71 infants (4.2%) (monoparesis *n* = 3; hemiparesis *n* = 8; diplegia *n* = 14; tetraparesis *n* = 9). Deafness occurred in 8 (1%) and blindness in 2 (0.2%) infants. Some infants had composite outcomes (one had cerebral palsy, blindness, deafness and neuropsychological deficiency and two patients showed cerebral palsy and deafness) (Figure 4). Patients with severe FD had lower birth weight and GA, required advanced neonatal resuscitation, mechanical ventilation, and showed a higher rate of NEC, PDA, BPD, ROP and cerebral lesions (detected by cerebral ultrasound). Neonates without severe FD were more frequently breastfed at discharge from the NICU (Table 5). Severe FD was more common in infants with a lower GA (Table 1, Figure 3, Figure S2). Cerebral palsy was also more common in infants with a lower GA: 23 of 260 (8.8%) in group 1; 8 of 389 (2%) in group 2; and 3 of 153 (2%) in group 3 (*p* < 0.001). In the univariate regression analysis, multiple variables were associated with severe FD (Table 6). The final multivariate regression model included five variables: GA, male gender, advanced neonatal resuscitation, PIH and PVL (area under ROC curve: 0.72).

**TABLE 4:** Moderate, severe and overall disability according to gestational age.

Gestational age (weeks)	Moderate disability (%)	Severe disability (%)	Global disability (%)
23	2/8 (25)	2/8 (25)	4/8 (50)
24	5/26 (19.2)	7/26 (26.9)	12/26 (46.1)
25	10/55 (18.2)	12/55 (21.8)	22/55 (40)
26	10/56 (17.9)	6/56 (10.7)	16/56 (28.6)
27	11/59 (18.6)	9/59 (15.2)	29/59 (33.8)
28	10/56 (17.8)	7/56 (12.5)	17/56 (30.3)
29	18/146 (12.3)	9/146 (6.2)	27/146 (18.5)
30	7/128 (5.5)	7/128 (5.5)	14/128 (11.0)
31	5/115 (4.3)	5/115 (4.3)	10/115 (8.6)
32	4/68 (5.9)	3/68 (4.4)	7/68 (10.3)
33	2/49 (4.1)	2/49 (4.1)	4/49 (8.2)
34	1/26 (3.8)	2/26 (7.7)	3/26 (11.5)
35	0/10 (0)	0/10 (0)	0/10 (0)
All	85/802 (10.6)	71/802 (8.9)	156/802 (19.4)

**TABLE 5:** Comparison between infants with or without severe functional disability at 24 months.

	Patients with severe functional disability	Patients without severe functional disability	*p*
	*N* = 71	*N* = 731	
Birth weight, median (IQR), g	1000 (720–1280)	1145 (900–1370)	**0.002**
Gestational age, median (IQR), weeks	28 (25.71–30.14)	29.4 (27.71–31.29)	**0.003**
Outborn, *n* (%)	3 (4.2)	18 (2.5)	0.348
Body temperature admission, median (IQR), °C	36.1 (35.6–36.5)	36.2 (35.9–36.6)	0.176
Multiple gestation, *n* (%)	22 (31.0)	228 (31.2)	0.902
Advanced neonatal resuscitation, *n* (%)	28 (39.4)	144 (19.6)	**< 0.001**
Apgar 5th min, median (IQR)	8 (7–9)	9 (8–9)	0.067
Caesarean delivery, *n* (%)	13 (18.3)	134 (18.3)	0.914
Male gender, *n* (%)	43 (60.6)	341 (46.6)	**0.012**
Prenatal steroids exposure, *n* (%)	64 (90.1)	657 (89.9)	0.474
Prenatal magnesium sulphate exposure, *n* (%)	18 (25.3)	246 (33.6)	0.258
Chorioamnionitis, *n* (%)	25 (35.2)	152 (21.0)	**0.003**
Surfactant administration, *n* (%)	43 (60.5)	359 (49.1)	**0.034**
Oxygen on day 28, *n* (%)	40 (56.3)	261 (40.53)	**0.004**
BPD, *n* (%)	21 (29.6)	124 (35.7)	**0.006**
Steroids administration in BPD, *n* (%)	24 (33.8)	101 (13.8)	**< 0.001**
Sepsis, *n* (%)	16 (22.5)	62 (8.5)	**< 0.001**
EOS, *n* (%)	2 (2.8)	16 (2.2)	0.703
LOS, *n* (%)	15 (21.1)	48 (6.6)	**< 0.001**
Mechanical ventilation, *n* (%)	43 (60.5)	243 (33.2)	**< 0.001**
Periventricular‐intraventricular haemorrhage, *n* (%)	35 (49.3)	282 (39.11)	**0.027**
Periventricular leukomalacia, *n* (%)	10 (14.1)	8 (1.11)	**< 0.001**
PDA, *n* (%)	36 (50.7)	266 (38.6)	**0.009**
PDA pharmacological treatment, *n* (%)	25 (35.2)	162 (22.2)	**0.008**
NEC, *n* (%)	7 (9.8)	7 (0.9)	**< 0.001**
NEC surgical treatment, *n* (%)	11 (15.5)	11 (1.5)	**< 0.001**
ROP surgical treatment, *n* (%)	9 (12.7)	31 (4.2)	**0.001**
Human milk feeding at discharge, *n* (%)	32 (49.3)	502 (68.7)	**0.005**

*Note:* The value in bold are statistically significant (*p* < 0.05).

Abbreviations: BPD, bronchopulmonary dysplasia; EOS, early‐onset sepsis; LOS, late‐onset sepsis; NEC, necrotizing enterocolitis; PDA, patent ductus arteriosus; PIH, periventricular–intraventricular haemorrhage; PVL, periventricular leukomalacia; ROP, retinopathy of prematurity.

**TABLE 6:** Perinatal data and severe functional disability at 24 months of corrected age.

	Univariate model			Multivariate model		
	OR	95% CI	*p*	OR	95% CI	*p*
Birth weight	0.99	0.99–0.99	**0.001**			
Gestational age	0.84	0.76–0.92	**< 0.001**	0.75	0.65–0.91	**0.035**
Outborn	1.80	0.52–6.29	0.355			
Body temperature admission	0.94	0.74–1.19	0.614			
Multiple gestation	1.03	0.61–1.76	0.902			
Advanced neonatal resuscitation	2.65	1.59–4.42	**< 0.001**	1.92	1.05–3.51	**0.034**
Apgar 5th min	0.88	0.75–1.01	0.086			
Caesarean delivery	1.03	0.55–1.95	0.914			
Male gender	1.92	1.15–3.20	**0.013**	1.84	1.06–3.21	**0.031**
Prenatal steroids exposure	1.46	0.51–4.15	0.477			
Prenatal magnesium sulphate	0.72	0.41–1.27	0.260			
Chorioamnionitis	2.15	1.27–3.64	**0.004**			
All sepsis	3.27	1.76–6.07	**< 0.001**			
EOS	1.33	0.30–5.93	0.704			
LOS	3.97	2.08–7.55	**< 0.001**			
Oxigen on Day 28	2.10	1.26–3.48	**0.004**			
Mechanical ventilation	3.38	2.02–5.67	**< 0.001**			
BPD	2.23	1.25–3.98	**0.007**			
Steroids administration in BPD	3.35	1.95–5.74	**< 0.001**			
PIH	1.76	1.06–2.91	**0.029**			
PIH grade	2.09	1.69–2.58	**< 0.001**	1.74	1.36–2.23	**< 0.001**
PDA	1.93	1.17–3.17	**0.010**			
PDA pharmacological treatment	2.01	1.19–3.38	**0.009**			
NEC	4.75	1.90–11.90	**0.001**			
NEC surgical treatment	12.46	5.17–29.98	**< 0.001**			
ROP surgical treatment	3.39	1.54–7.47	**0.002**			
Surfactant administration	1.73	1.04–2.90	**0.036**			
Periventricular leukomalacia	15.89	6.03–41.87	**< 0.001**	9.94	3.28–30.14	**< 0.001**
Human milk feeding at discharge	0.49	0.30–0.81	**0.005**			

*Note:* The values in bold are statistically significant (*p* < 0.05).

Abbreviations: BPD, bronchopulmonary dysplasia; EOS, early‐onset sepsis; LOS, Late‐onset sepsis; NEC, necrotizing enterocolitis; PDA, patent ductus arteriosus; PIH, periventricular–intraventricular haemorrhage; PVL, periventricular leukomalacia; ROP, retinopathy of prematurity.

Moderate FD occurred in 85/802 (10.6%) infants. Thirty‐two of 85 patients (37.6%) presented Bayley III cognitive composite score ≤ 1 DS (18 cases) or GMDS‐R global development quotient ≤ 1 DS (14 cases); 3 (3.5%) had unilateral blindness and 50 (58.8%) cases presented neuromotor abnormalities. Moderate FD was more common in infants with a lower GA (Table 1, Figure 3 and Figure S2). Overall, 282 of 802 infants (35.2%) were sent to neuromotor rehabilitation during the first 24 months of life, more frequently if they had a lower GA: 141 of 260 (54.2%) in group 1; 116 of 389 (29.8%) in group 2; and 25 of 153 (16.3%) in group 3 (*p* < 0.001).

The GMDS‐R subscales, GMDS‐R global quotient and Bayley III composite scores were compared among the three GA groups. The GMDS‐R global development quotient, locomotor, personal and social, hearing and language, eye and hand coordination quotient as well as the Bayley III cognitive and language composite scores differed significantly among groups. Patients of Group 1 had a lower score than the others (Table 7).

**TABLE 7:** Comparison of GMDS‐R quotients or BSDI III scores among three groups of different gestational ages.

		Group 1	Group 2	Group 3	*p*
		*N* = 260	*N* = 389	*N* = 153	
Patients evaluated with GMDS – R *N* = 363	**GMDS – R**	**GMDS – R**	**GMDS – R**	**GMDS – R**	
	Global quotient	90.7 ± 15.3	98.8 ± 13.6	100 ± 11.6	**< 0.001**
	Locomotor quotient	92.8 ± 16.9	103 ± 17.2	102 ± 16.3	**< 0.001**
	Personal and social quotient	92.7 ± 17.2	101 ± 15.5	103 ± 15.3	**< 0.001**
	Hearing and language quotient	87.1 ± 17.7	94.4 ± 16.6	95.5 ± 14.1	**< 0.001**
	Eye and Hand Coordination quotient	95.9 ± 16.8	103 ± 14.5	103 ± 12.4	**< 0.001**
	Performance quotient	93.7 ± 15.2	99.6 ± 15.3	98.4 ± 14.6	**< 0.001**
Patients evaluated with Bayley III *N* = 439	**Bayley–III**	**Bayley– III**	**Bayley– III**	**Bayley III**	
	Cognitive composite score	90.1 ± 11.6	95.7 ± 9.25	99.6 ± 11.6	**< 0.001**
	Motor composite score	89.3 ± 13.0	94.1 ± 9.12	98.2 ± 11.2	0.0052
	Language composite score	85.5 ± 12.4	90.6 ± 9.84	92.7 ± 10.3	**< 0.001**

*Note:* Group 1: ≤ 28 weeks' gestation; Group 2: > 28–31 weeks' gestation; Group 3: ≥ 32 weeks' gestation.

## 4 Discussion

This multicentric study provided valuable insights into mortality rates, neurodevelopmental outcomes, and associated predictors among a cohort of VLBW infants born in Italy. The findings contributed to the growing body of literature on neonatal outcomes in this vulnerable population, highlighting key risk factors and potential areas for intervention. The mortality rates observed in this cohort aligned with previous studies on VLBW infants, emphasising the continued challenges in improving survival outcomes. Large cohort studies reported mortality rates between 10%–15% in VLBW infants, depending on GA, birth weight and neonatal interventions [14, 15, 16, 17, 18, 19, 20, 21, 22]. We found that 9.8% of VLBW infants died during their hospital stay, with GA emerging as a major predictor of mortality. This finding is consistent with previous studies [14, 15, 16, 17, 18, 19, 20, 21, 22]. Interestingly, mortality affected 100% of babies born at 22 weeks, decreased to 75% at 23 weeks and dropped drastically after 24 weeks. Although updated Italian guidelines on resuscitation at the threshold of viability are currently lacking, a prior consensus, still considered valid, recommends a nuanced and individualised approach for newborns at 22 weeks gestational age. This guidance emphasises careful clinical assessment, active parental involvement in decision‐making, and a personalised evaluation considering gestational age, associated risk factors and potential outcomes. Such a cautious strategy, rather than a uniformly proactive approach, may partly explain the observed 100% mortality rate at 22 weeks in our cohort. It is important to note, however, that the sample size for this GA group was very small, limiting the generalisability of this finding [23]. This highlights the need for cautious interpretation of mortality data at the threshold of viability. Anyway, in our study, mortality decreased as GA increased, reflecting improved survival chances for infants born at later gestational weeks. This finding was consistent with a previous meta‐analysis, showing that mortality risk reduced for each additional weeks' gestation [19]. Furthermore, prenatal administration of magnesium sulphate and corticosteroids was protective against mortality, in line with a systematic review on prenatal magnesium sulphate administration [20]. Magnesium sulphate has been shown to reduce the risk of intraventricular haemorrhage and cerebral palsy, while steroids improved lung maturation and reduced respiratory distress syndrome [20]. In contrast, advanced resuscitation, defined as intubation in the delivery room, was strongly associated with increased neonatal mortality. This finding probably reflected the fact that infants requiring intubation at birth were those with significant cardiorespiratory compromise, often due to extreme prematurity itself, perinatal asphyxia, or other complications. Similarly, admission temperature was significantly associated with neonatal mortality, highlighting the critical importance of effective thermal management immediately after birth to reduce the risk of death. Like previous studies, our data confirmed that EOS, NEC and PDA were also significantly associated with mortality [21, 22]. Our multivariate model showed strong predictive capability for mortality risk, indicating robust associations (high predictive accuracy with AUC = 0.91). This suggests our findings were externally valid and representative of global trends in the NICUs.

Regarding the prevalence of neurodevelopmental impairments, we found severe FD in 8.9% and moderate FD in 10.6% of infants who completed follow‐up. It is interesting to note that disability primarily affected infants born before 26 weeks, followed by a significant decline. This trend was evident in both disability and mortality rates, which decreased significantly with advancing GA. Infants born before 28 weeks experienced the highest mortality (19.4%) and a substantial burden of moderate (18.5%) and severe (16.5%) disabilities among survivors, with only 65% surviving without disability. In contrast, outcomes improved markedly after 28 weeks, with minimal mortality and over 90% of infants born at 32 weeks or later surviving without disability. This finding highlighted the critical impact of gestational maturity on neurodevelopmental outcomes. However, during follow‐up, 35.2% of infants showed neurological abnormalities requiring rehabilitation. These results were consistent with existing studies, which reported severe disability rates ranging from 6% to 12% and moderate disability rates between 10% and 20% in VLBW survivors [9, 24, 25]. Anyway, our findings suggested a slightly lower prevalence of cerebral palsy and sensory disabilities compared to some previous studies, potentially reflecting advances in neonatal care, such as improved perinatal neuroprotection through magnesium sulphate and antenatal steroids, as well as better management of neonatal brain injuries with optimised ventilation and early neurodevelopmental interventions [26]. Our study identified GA, male gender, PVL, PIH and advanced neonatal resuscitation as independent predictors of severe FD. These are well‐established risk factors in preterm neurodevelopmental outcomes [25, 26]. Our findings support the hypothesis that male preterm infants have higher rates of neurodevelopmental impairments due to differences in brain maturation and vulnerability to inflammation [27]. Moreover, severe perinatal distress and hypoxic–ischaemic injury increased disability risk, in agreement with recent perinatal studies [19, 21, 26]. PVL and PIH also resulted strongly associated with cerebral palsy and motor dysfunction, confirming the important role of cerebral lesion in determining the outcome [19, 21, 26]. Furthermore, our study found that infants born ≤ 28 weeks GA had the worst neurodevelopmental scores across multiple domains, including motor, cognitive and social skills. This finding aligned with previous large‐scale studies, which reported that extremely preterm infants (< 27 weeks) had significantly lower cognitive and motor scores compared to later preterm [8, 9, 15, 16, 25, 28]. Breastfeeding at NICU discharge was associated with better neurodevelopmental outcomes, confirming prior evidence on the protective role of human milk for preterm brain development [29, 30]. Our data reinforce the importance of prenatal magnesium sulphate and antenatal steroids for improving survival and reducing disability. Strategies to prevent chorioamnionitis and foetal inflammation may further reduce perinatal brain injury. Minimising mechanical ventilation and promoting early extubating could reduce lung and brain injury risks. Enhanced sepsis prevention strategies, including antibiotic stewardship and early skin‐to‐skin care may improve survival and neurodevelopment. Our study highlights the high burden of neurodevelopmental impairment, emphasising the need for early rehabilitation programmes, particularly for infants born < 27 weeks. Targeted follow‐up programmes for high‐risk neonates, such as extreme preterm or those with PVL, cerebral palsy or cognitive deficits, can improve long‐term outcomes.

### 4.1 Strengths and Limitations

One of the major strengths of the study was the comprehensive 24‐month neurodevelopmental follow‐up. Unlike studies that focus solely on in‐hospital survival, this study extended its scope to include neurological impairments at 24 months, providing a more complete picture of the challenges faced by these infants. By analysing a large cohort of 1381 infants, it offered robust statistical power and meaningful conclusions. A key methodological strength was the use of multivariate logistic regression models to identify independent predictors of mortality and neurodevelopmental impairment. The high area under the curve (AUC = 0.91) in the mortality prediction model demonstrates its strong predictive accuracy. The study examined critical perinatal factors, such as antenatal steroid and magnesium sulphate administration, neonatal complications and mechanical ventilation, all of which had been shown in previous literature to impact outcomes. Furthermore, the study's GA stratification highlighted how developmental impairments differ based on the degree of prematurity. This kind of subgroup analysis is essential for tailoring neonatal care and early intervention strategies to infants at the highest risk. Despite these strengths, some limitations must be acknowledged. One of the primary concerns was the loss to follow‐up. Only 802 of the 1224 eligible infants (65.5%) completed the 24‐month assessment. Contributing factors likely included relocation, logistical difficulties and personal circumstances. Furthermore, the COVID‐19 pandemic exacerbated barriers to follow‐up during the study period. In addition, some infants may have been partially assessed but did not complete the full follow‐up protocol, or their data may not have been properly recorded. Although follow‐up was primarily conducted through hospital‐based outpatient clinics, some children may have been evaluated in other facilities, such as habilitation centers, without systematic data sharing, leading to incomplete outcome capture. This attrition may have introduced selection bias, potentially leading to underrepresentation of children with milder impairments, those from disadvantaged backgrounds, or those with severe disabilities managed outside our system. Moreover, while the study provided valuable short‐term neurodevelopmental data, assessing outcomes at 24 months did not fully predict cognitive and motor function at school age. Many neurodevelopmental impairments, especially in cognitive and executive functioning, became more evident at 5–7 years rather than in early childhood. The study also did not deeply account for socioeconomic and environmental factors, which are known to influence neurodevelopmental trajectories in preterm infants. Additionally, although the study included a broad range of GA, extremely preterm infants (less than 25 weeks of gestation) were underrepresented, meaning the findings could not fully reflect the unique challenges faced by these infants. Future research should prioritise targeted recruitment and tailored follow‐up protocols for extremely preterm infants to ensure their adequate representation. Implementing strategies such as centralised data registries and enhanced communication with families could improve data completeness and compliance with follow‐up visits. These approaches may have facilitated a more comprehensive understanding of outcomes in this high‐risk population and supported the development of optimised care pathways.

## 5 Conclusion

In conclusion, the study significantly contributed to the understanding of mortality and neurodevelopmental impairment in VLBW infants, aligning with global research trends. A complex interplay of medical and perinatal factors influenced the survival and neurodevelopmental outcomes of an Italian cohort of VLBW infants.

Continued advancements in neonatal care, along with targeted early intervention strategies, were deemed essential for improving both short‐ and long‐term outcomes. Moreover, the findings served as a valuable tool in prenatal counselling by providing evidence‐based information to support parents facing the risk of preterm birth and assisting healthcare professionals in guiding shared decision‐making.

Future research should focus on long‐term follow‐up and the evaluation of intervention programmes aimed at enhancing the quality of life for this high‐risk population. To ensure the validity and completeness of outcome assessments, minimising loss to follow‐up remained a critical priority.

## Conflicts of Interest

The authors declare no conflicts of interest.

## Supporting information


**Data S1:** apa70292‐sup‐0001‐Supinfo.docx.

## Data Availability

The data that support the findings of this study are available on request from the corresponding author. The data are not publicly available due to privacy or ethical restrictions.
