# Supplementary material for: Mortality and Neurodevelopmental Outcome in an Italian Cohort of Very Low Birth Weight Infants
Source: Acta Paediatr. 2025 Aug 27;115(1):153–65. doi: 10.1111/apa.70292 (PMC12687702; doi:10.1111/apa.70292)
Supplement: Supplementary file 1 — Data S1: apa70292‐sup‐0001‐Supinfo.docx. [file APA-115-153-s001.docx]

**Figure S1 – Surviving and deceased infants according to gestational age**

**Figure S2 – Patients with severe and moderate functional disability according to gestational age at birth.**
